# Psychometric properties of the Latino Students Patient Safety Questionnaire, Brazilian version

**DOI:** 10.1590/0034-7167-2021-0961

**Published:** 2023-02-06

**Authors:** Fabrícia Moreira Amorim Amaral, Aline Carrilho Menezes, Cissa Azevedo, André Carlos Santos Ferreira, Helen Cristiny Teodoro Couto Ribeiro, Luciana Regina Ferreira da Mata

**Affiliations:** I Universidade Federal de São João del-Rei. Divinópolis, Minas Gerais, Brazil; II Universidade Federal de Minas Gerais. Belo Horizonte, Minas Gerais, Brazil

**Keywords:** Patient Safety, Psychometrics, Health Knowledge, Attitudes, Practice, Students, Nursing, Students, Medical, Seguridad del Paciente, Psicometría, Conocimientos, Actitudes y Práctica en Salud, Estudiantes de Enfermería, Estudiantes de Medicina, Segurança do Paciente, Psicometria, Conhecimentos, Atitudes e Prática em Saúde, Estudantes de Enfermagem, Estudantes de Medicina

## Abstract

**Objectives:**

to assess the psychometric properties of the Latino Students Patient Safety Questionnaire, Brazilian version.

**Methods:**

a methodological study, carried out between April 2020 and January 2021, with 218 nursing and medicine students. Structural and discriminant construct validity were assessed by confirmatory factor analysis and cross factor loadings. Reliability was verified by McDonald’s omega, average variance extracted, composite reliability, and item-total correlation.

**Results:**

the final model reproduced the original structure of 21 items, distributed in five dimensions, requiring the exclusion of one item. Acceptable fit indexes were obtained (x^2^/gl=2.325; CFI=0.99; TLI=0.98; RMSEA=0.054). Discriminant validity was confirmed. Reliability indicators were adequate, except McDonald’s omega, in one factor (0.68), and average variance extracted, in two factors (0.41; 0.47).

**Conclusions:**

the instrument demonstrated evidence of internal validity and satisfactory reliability among nursing and medical students.

## RESUMO

**Objetivos:**

avaliar as propriedades psicométricas da versão brasileira do *Latino Students Patient Safety Questionnaire.*

**Métodos:**

estudo metodológico, realizado entre abril de 2020 e janeiro de 2021, com 218 estudantes de graduação em enfermagem e medicina. Validades de construto estrutural e discriminante foram aferidas pela análise fatorial confirmatória e cargas fatoriais cruzadas. A confiabilidade foi verificada pelo ômega de McDonald, variância média extraída, confiabilidade composta e correlação item-total.

**Resultados:**

o modelo final reproduziu a estrutura original de 21 itens, distribuídos em cinco dimensões, sendo necessária a exclusão de um item. Obtiveram-se índices de ajustes aceitáveis (x^2^/gl=2,325; CFI=0,99; TLI=0,98; RMSEA=0,054). Confirmou-se a validade discriminante. Os indicadores de confiabilidade foram adequados, exceto ômega de McDonald, em um fator (0,68), e variância média extraída, em dois fatores (0,41 ; 0,47).

**Conclusões:**

o instrumento demonstrou evidência de validade interna e confiabilidade satisfatória entre estudantes de enfermagem e medicina.

## RESUMEN

**Objetivos:**

evaluar las propiedades psicométricas de la versión brasilena Latino Students Patient Safety Questionnaire, versión brasilena.

**Métodos:**

estudio metodológico, realizado entre abril de 2020 y enero de 2021, con 218 estudiantes de pregrado en enfermería y medicina. La validez de constructo estructural y discriminante se evaluó mediante análisis factorial confirmatoria y cargas factoriales cruzadas. La confiabilidad fue verificada por el omega de McDonald, Ia varianza promedio extraída, Ia confiabilidad compuesta y la correlación ítem-total.

**Resultados:**

ei modelo final reprodujo Ia estructura original de 21 items, distribuídos en cinco dimensiones, requiriendo la exclusión de un ítem. Se obtuvieron índices de ajuste aceptables (x^2^/gl=2,325; CFI=0,99; TLI=0,98; RMSEA=0,054). Se confirmó la validez discriminante. Los indicadores de fiabilidad fueron adecuados, excepto el omega de McDonald, en un factor (0,68), y la varianza media extraída, en dos factores (0,41; 0,47).

**Conclusiones:**

el instrumento demostró evidencias de validez interna y confiabilidad satisfactoria entre estudiantes de enfermería y medicina.

## INTRODUCTION

Patient Safety (PS) is recognized as one of the components of quality of care^(1)^ and a priority in health services^(2)^. It is conceptualized as a range of organized activities aimed at developing strategies that guide organizational culture for promoting safe environments and reduction of occurrence of avoidable damage and its impacts^(2)^.

Including the topic in the scope of professional education, with a view to improving quality and strengthening safety culture, is an international recommendation^(3-4)^. In Brazil, its insertion in the curricula of health training courses has been determined since the publication of the Brazilian National Patient Safety Program (*Programa Nacional de Segurança do Paciente*) in 2013^(5)^.

In order to qualify the teaching of the topic, scholars^(6)^ elaborated guidelines in order to describe, in the context of multidisciplinary education, knowledge, skills, attitudes and behaviors that health professionals must develop to provide safe care. These guidelines also guided the construction of the World Health Organization Patient Safety Curriculum Guide: Multi-professional Edition^(7)^, especially with regard to the inclusion of the topic in teaching within the health area.

Teaching about safe practices is considered a challenge^(2)^ involving both educators and educational institutions. Although there is no consensus on how to approach the topic during the training of health professionals, continuous and transversal teaching is recommended, from the early years, in order to promote the development of skills for PS^(8)^.

In this regard, knowledge and attitude assessment in PS among students in the health area is a relevant strategy to identify weaknesses related to teaching on the subject, which will direct adjustments to Pedagogical Political Projects as well as adjustments in teaching methodologies that favor topic integration into the curricula^(3-4)^. Moreover, this type of approach allows students to reflect on what they need to learn on the subject, what skills they need to acquire in their training, and this, consequently, will reflect on quality of care and safety culture strengthening^(9)^. With regard to assessment of PS-related competencies, it is recommended that this be carried out through valid and reliable instruments that reflect the education scenario and that can guide the educational institution in proposing actions to promote knowledge about PS^(4,10)^.

In a literature search, 19 questionnaires were identified that assess competencies and/or perception scans of PS among undergraduate students in health. However, only two instruments were cross-culturally adapted into Brazilian Portuguese: the Health Professional Education in Patient Safety Survey (H-PEPSS)^(11)^ and the Latino Students Patient Safety Questionnaire (LSPSQ)^(12)^. No studies were identified to assess the psychometric properties in the Brazilian context for these instruments, which allows us to analyze how much an instrument actually measures what it proposes to measure and how much it is free of measurement errors^(13)^ so that researchers can have more confidence in the results obtained.

The LSPSQ was elaborated with the objective of assessing knowledge and attitudes about PS among Latin undergraduate students in medicine and nursing inserted in clinical practice. It is an instrument of 21 items, distributed in five dimensions, and assessed by a Likert-type scale^(14)^. The choice of this instrument is based on the audience for which it was structured and on the greater correspondence and proximity to the reality of health education in Brazil.

In a previous study^(12)^, translation and cross-cultural adaptation of LSPSQ was performed. Thus, the present study intends to verify the psychometric properties of this version, in order to provide a valid and reliable instrument, which can be applied to the context of Brazilian students, for the identification of knowledge and attitudes about PS. It is expected that the use of this instrument will make it possible to identify the needs to approach the topic among medical and nursing students in Brazil and contribute to the insertion and strengthening of PS during the training of health professionals. Thus, the research question was: is the LSPSQ a valid and reliable instrument to verify knowledge and attitudes of undergraduate medical and nursing students?

## OBJECTIVES

To assess the LSPSQ's psychometric properties, Brazilian version.

## METHODS

### Ethical aspects

The study was approved by the proposing institution’s Research Ethics Committee (REC). Universities related to data collection were included as co-participants. All participants were aware of and agreed to the Informed Consent Form (ICF), according to Resolution 466/2012 of the Brazilian National Health Council.

### Study design, period and site

This is a cross-sectional methodological study. The assessment of the instrument’s psychometric properties was based on the COnsensus-based Standards for the selection of health Measurement INstruments (COSMIN) recommendations, based on the use of a checklist for assessment studies of measurement properties^(13,15)^.

Cross-cultural adaptation^(12)^ and assessment of the LSPSQ’s psychometric properties were conducted with the authorization of the authors of the original version of the instrument^(14)^. Information presentation was conducted based on the Strengthening the Reporting of Observational Studies in Epidemiology (STROBE) guidelines^(16)^.

When considering the territorial extension of Brazil and cultural diversity, a sample with representation from different Brazilian regions was sought. To this end, an internet survey was conducted of federal and state public universities that had nursing and medicine courses. An e-mail with presentation of the project and invitation to participate was sent to the coordinators of these courses. Of the 57 universities, 29 agreed to participate in the study. Among these, a selection was made for convenience, considering those with the highest number of students enrolled and that offered both courses on the same campus, to speed up the process of including these institutions as co-participants in the research. Thus, data were collected on 14 campuses of 11 public universities, located in the Northeast (n=05), Southeast (n=03), Midwest (n=02) and South (n=01 ) regions of Brazil, between April 2020 and January 2021.

### Population: sample definition and inclusion and exclusion criteria

Sample size was defined based on the methodological recommendations proposed by COSMIN^(15)^, for conducting factor analysis, which establishes a minimum of seven observations for each item of the instrument An invitation to participate was sent to 1,093 students, enrolled in the last year of an undergraduate nursing course (n=423) or in the last two years of graduation in medicine (n=670), who had already experienced an internship in a hospital area. The number of responses obtained was 255 (23.33%). Participants who did not complete the questionnaire (n=37) were excluded, totaling a sample of 218 valid answers (137 nursing and 81 medicine).

### Study protocol

Through the course coordinators of the participating universities, the researchers had access to the list of students and their e-mail addresses. Data collection was structured on a free online platform called e-Surv. The link to access the instrument was forwarded with the invitation to participate via e-mail. Every seven days, a new email was sent to non-responder students, for up to five attempts, or until the minimum number of responses was obtained.

The instrument sent to participants was the LSPSQ^(12)^, translated and adapted cross-culturally into Portuguese Brazilian. The questionnaire is self-administered, contains 21 statements distributed in five dimensions: *franqueza na comunicação* (FCP): eight statements; *atitude proativa para evitar risco à segurança* (AP): four statements; *consciência do erro* (CE): three statements; *compreensão do fator humano* (CFH): four statements; *complexidade dos sistemas e sua interrelação* (CS): two statements. Each of these statements is assessed using a Likert scale, ranging from one (totally disagree) and five (totally agree), and statements CS1 and CS2 were written negatively and should have the values of scores inverted^(14)^. Each dimensions is assessed according to the average score of the statements that compose it. Scores equal to or greater than four points indicate that students had the opportunity to acquire the expected competency in PS^(17)^.

In the validity study of the original version^(14)^, the instrument showed adequate internal consistency, with Cronbach’s alpha greater than 0.80 for the complete questionnaire and greater than 0.70 for the dimensions, except for the CS dimension (0.67). The item-total correlations were higher than 0.30 in all cases. In convergent-discriminant validity analysis, all standardized loads were considered significant for the respective factor with values greater than 0.60. The results showed a good fit of the model with the Goodness of Fit Index (GFI) and the Adjusted Goodness of Fit Index (AGFI) of 0.90^(14)^.

The instrument is also composed of questions related to sociodemographic characterization, with the following variables: sex; age; city and state of origin; undergraduate course and period; university; hospital internship experience; number of beds in the hospital in which internship takes place; current stage field; teacher monitoring during internship activities; other training in the health area; employment relationship in a health unit; and approach to PS throughout the course^(14)^.

### Analysis of results, and statistics

For data analysis, the Lavaan package of the R software, version 4.0.2, was used. First, a descriptive analysis was performed for participant characterization. The existence of univariate outliers was verified, by the standardization of results, and of multivariate outliers, by the D^2^ measure of Mahalanobis. Normality was also verified using the Shapiro-Wilk test and linearity using Bartlett’s test of sphericity.

To verify construct validity evidence, aspects related to structural validity were assessed. Confirmatory factor analysis (CFA) can be used, among many other objectives, to verify if the factor structure of a proposed model reflects the theoretical concept of the construct, when applied to a given sample. Since it is a confirmatory test, this factorial structure must be previously defined by the researcher at the time of the test^(18)^. Thus, CFA was performed to verify the psychometric structure plausibility of the LSPSQ, Brazilian version^(12)^, whose structure was already predefined^(14,13)^. Dimensionality was ascertained by the Kaiser criterion so that each factor must be equal to one^(18)^. Analysis was implemented using a polychoric matrix and the Robust Diagonally Weighted Least Squares (RDWLS) estimation method, suitable for categorical data. Initially, it was necessary to verify the factor loadings of each item, since items with values lower than 0.50 do not contribute significantly to the formation of the latent variable, and, therefore, their exclusion must be assessed^(18)^.

CFA can also be used to verify discriminant validity, which concerns how much a construct differs from others^(18)^. In this context, discriminant validity was verified by the criterion of cross loadings, being obtained when the factor loading of all its items is greater than the respective cross factor loadings^(18)^.

To assess the model’s goodness of fit, we used chi-square statistics (χ^2^); chi-square ratio by degrees of freedom (χ^2^/gl), Comparative Fit Index (CFI), Tucker-Lewis Index (TLI) and Root Mean Square Error of Approximation (RMSEA). χ^2^ values should not be significant and the χ^2^/gl ratio should preferably be < three^(19)^. CFI and TLI values should preferably be ≥ 0.95^(19)^. RMSEA values should preferably be < 0.06^(19)^, and the upper limit of the confidence interval should be < 0.10^(19)^.

For reliability analysis, the internal consistency of the instrument for which the use of McDonald’s omega is recommended was assessed. It was also decided to use composite reliability (CR), average variance extracted (AVE) and item-total correlation assessment. The use of these four indicators aimed to improve reliability interpretation. As an interpretation parameter, McDonald’s omega and/or CR indicators must present values ≥ 0.70^(18)^. For AVE, values must be ≥ 0.50^(18)^. For item-total correlation, values must be ≥ 0.50^(18)^.

## RESULTS

Table 1 shows sociodemographic characteristics of 218 study participants. The database included 38 variables, 17 of which were sociodemographic and the others related to the items. Thus, 444 missing data were found that referred to the characterization variables.

**Table 1:** Sociodemographic characteristics of nursing (N=137) and medicine (N=81 ) students, Divinópolis, Minas Gerais, Brazil, 2019-2020

Variables	Nursing		Medicine	
	n	%	n	%
Sex				
Female	105	76.64	49	60.49
Male	32	23.36	32	39.51
Age in years	24.72*	3.83^†^	25.5*	3.28^†^
Academic profile				
9^th^ period	94	68.61	24	29.63
10^th^ period	29	21.17	28	34.57
11^th^ period	-	-	20	24.69
12^th^ period	-	-	9	11.11
Residence region				
Northeast	56	40.88	23	28.40
Southeast	66	48.18	51	62.96
Midwest	10	7.30	-	-
South	5	3.65	7	8.64
Monitoring of curricular internship supervisor during activities				
Yes	84	61.31	63	77.78
In part	46	33.58	17	20.99
No	7	5.11	1	1.23
Frequency of approaching the patient safety topic during the course				
Always	104	75.91	25	30.86
Few times	32	23.36	52	64.20
Never	1	.73	4	4.94

*average age of participants; †standard deviation of age of participants.

In the analysis of outliers, no value was found outside the range of the scale of its respective variable so that no outlier related to error in data tabulation was evidenced. Moreover, according to analysis criteria, no univariate or multivariate observation considered atypical was found.

Normality analysis showed that the LSPSQ items did not show normal distribution. In data linearity analysis, Bartlett’s sphericity test showed significant results, with p ≤ 0.05 in all factors. These results allowed using CFA as an adequate technique to interpret the information contained in this matrix.

Table 2 presents the factor loadings of each item, obtained from conducting CFA. Although FCP1 had a factor loading of less than 0.50, it was maintained due to its relevance to the research and for not having a negative impact on the initial model’s adjustment indexes. FCP8, on the other hand, had a factor loading of 0.36, and given its theoretical scope to address patient safety culture and institutional attitudes, we opted for its exclusion. The initial model’s factor loadings were presented, obtained from CFA performed with all the final model and instrument items, which represents the result obtained after the exclusion of FCP8 (Table 2). All factors were unidimensional by the Kaiser criterion, with results equal to one. Regarding discriminant validity assessment, all items of each factor presented factor loadings higher than their respective cross factor loadings, being verified only for the final model (Table 2).

**Table 2:** Confirmatory factor analysis of the Latino Students Patient Safety Questionnaire, Brazilian version, initial and final model, Divinópolis, Minas Gerais, Brazil, 2019-2020

Dimensions/item	Initial model	Final model	
	λ	λ	Max. CFL
*Franqueza na comunicação (FCP)*			
*FCP1 : Aprendi a forma correta de fornecer informações aos pacientes que sofreram algum dano ou lesão por causa de um erro.*	.45	.43	.31
*FCP2: Durante o estágio, aprendi a avaliar os riscos que podem comprometer a segurança do paciente.*	.87	.87	.72
*FCP3: No estágio, aprendi o que devo fazer se eu cometer um erro.*	.63	.63	.54
*FCP4: Durante o estágio, tive a oportunidade de discutir com meus tutores ou preceptores qualquer condição de insegurança que eu pudesse ter observado.*	.63	.63	.53
*FCP5: Durante minha formação, adquiri competências sobre como relatar corretamente um erro aos meus colegas e aos meus superiores.*	.66	.65	.52
*FCP6: Durante minha formação, fui trabalhando os sentimentos que posso vir a vivenciar se eu cometer um erro.*	.57	.57	.44
*FCP7: Aprendi como me comunicar melhor com os pacientes para evitar erros de medicação.*	.71	.70	.55
*FCP8: No hospital onde fiz meu estágio, promovia-se uma cultura não punitiva, para que, caso ocorresse um erro, soubéssemos como evitar que ele se repetisse.*	.36	-	-
*Atitude proativa para evitar risco à segurança (AP)*			
*AP1: Durante meus estudos, recebi explicações sobre o que devo fazer para evitar os erros mais frequentes e garantir a segurança do paciente*	.86	.85	.69
*AP2: Durante o estágio, aprendi que, quando acontece um erro, devem ser tomadas medidas para que não ocorra novamente.*	.72	.72	.58
*AP3: Os professores discutem em sala de aula os erros mais comuns de serem cometidos e nos apontam formas de evitá-los.*	.81	.81	.66
*AP4: Durante minha formação, os professores nos explicaram os objetivos e as prioridades para tornar a assistência à saúde mais segura.*	.86	.86	.70
*Consciência do erro (CE)*			
*CE1: Durante meu estágio, pelo menos em uma ocasião, fiz algo que não era seguro para o paciente.*	.76	.76	.07
*CE2: Durante meu estágio, vi um colega fazer algo que não era seguro para o paciente.*	.76	.76	.06
*CE3: Durante meu estágio, vi profissionais fazerem algo que não era seguro para o paciente.*	.76	.76	.30
*Compreensão do fator humano (CFH)*			
*CFH1: Nos serviços de saúde, em que realizei meus estágios, me explicaram as normas de segurança adotadas para os pacientes.*	.51	.51	.33
*CFH2: Os professores enfatizaram a importância de seguir os protocolos para uma melhor assistência à saúde.*	.81	.80	.53
*CFH3: Durante o estágio, foi enfatizada a importância, para a segurança do paciente, de se fazer uso adequado dos recursos terapêuticos.*	.80	.81	.50
*CFH4: Os professores enfatizaram a importância de se lavar as mãos.*	.61	.62	.40
*A complexidade dos sistemas e sua interrelação (CS)*			
CS1: No estágio, observei que é impossível evitar a maioria dos erros clínicos.*	.76	.76	.24
CS2: Durante meu estágio, observei que os protocolos aplicados para garantir a segurança dos pacientes estão desatualizados (obsoletos).*	.76	.76	.20

λ - factor loading; Max. CFL - maximum cross factor loading; FCP - franqueza na comunicação; AP - atitude proativa; CE - consciência do erro; CFH - compreensão do fator humano; CS-a complexidade dos sistemas e sua interrelação; *inverted items.

The fit indexes supported the final model, except for the chi-square, which presented a significant result (p<0.001 ). The other results were χ^2^/gl=1.640, CFI=0.987, TLI=0.984 and RMSEA=0.054 (95% Confidence Interval (95% CI) =0.042-0.066).

**Figure 1:**
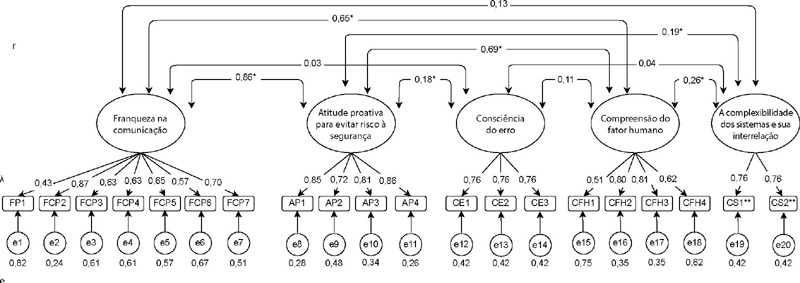
Path diagram of the Latino Students Patient Safety Questionnaire, Brazilian version, Divinópolis, Minas Gerais, Brazil, 2019-2020

Figure 1 shows the path diagram with the result of factor loadings, the correlation between dimensions (r) and the standard error of items (e).

r - correlation between factors; λ - factor loading; e - standard error; FCP - franqueza na comunicação; AP - atitude proativa; CE - consciência do erro; CFH - compreensão do fator humano; CS - a complexidade dos sistemas e sua interrelação;* p<0.05; **inverted items.

Regarding internal consistency (Table 3), assessed using McDonald’s omega, CR, AVE and item-total correlation, the values obtained in the AP, CE and CS dimensions were all satisfactory. In the FCP dimension, only AVE (0.41 ) was not considered adequate. In the CFH dimension, only the composite reliability (0.78) obtained a satisfactory value.

**Table 3:** Reliability measures of the Latino Students Patient Safety Questionnaire, Brazilian version, Divinópolis, Minas Gerais, Brazil, 2019-2020

Dimensions	Item	ω	CR	AVE	ITC
*Franqueza na comunicação (FCP)*	FCP1	.70	.83	.41	.63
	FCP2				.68
	FCP3				.78
	FCP4				.61
	FCP5				.78
	FCP6				.63
	FCP7				.61
*Atitude proativa para evitar risco à segurança (AP)*	AP1	.84	.89	.66	.82
	AP2				.61
	AP3				.87
	AP4				.85
*Consciência do erro (CE)*	CE1	.72	.80	.58	.87
	CE2				.86
	CE3				.71
*Compreensão do fator humano (CFH)*	CFH1	.68	.78	.47	.70
	CFH2				.74
	CFH3				.82
	CFH4				.53
*A complexidade dos sistemas e sua interrelação (CS)*	CS1	.88	.73	.58	.81
	CS2				.68

**ω** - *McDonald’s omega; CR - composite reliability; AVE - average variance extracted; ITC - item-total correlation.*

## DISCUSSION

The results of this study demonstrated construct and structural validity^(13,15,18-19)^ of the LSPSQ^(12)^, Brazilian version, for this sample. The structure obtained reproduced the original model^(14)^ regarding the number of factors and the distribution of items. However, it was necessary to reduce the measure in an item, which presented factorial load less than 0.50^(18)^. The final LSPSQ model was supported by excellent adjustment indexes^(19)^, and discriminant validity was confirmed^(18)^.

The results also indicate that instrument reliability^(13,15,18)^ was adequate. Although McDonald’s omega and VME values were lower than the recommended values in some factors, as already demonstrated, CR indexes were adequate in all factors as well as the item-total correlation indexes.

CFA is an analysis technique that allows verifying instrument structure in a given sample, item correlation in each dimension (factor loadings)^(19)^ as well as assessing the consonance of the model proposed with the literature^(18)^. Factor loading values are considered adequate when higher than 0.50 and as excellent above 0.70^(18)^ so that in this study most items in the model were higher than the latter.

CFA results indicated that the structure previously determined in the original instrument was confirmed when applied to the Brazilian context, remaining with five dimensions. FCP1, however, obtained a factorial load marginally below the recommended (0.45). It is understood that *“Aprendi a forma correta de fornecer informações aos pacientes que sofreram algum dano ou lesão por causa de um erro”* is directly related to the aspects of communication within the scope of PS, besides directing students to reflect on their knowledge and attitudes towards situations that require actions related to FCP. Thus, it was decided to maintain it in the final model, mainly because effective communication represents a strategy for preventing occurrence of adverse events^(20)^. In addition to this context, communication between professionals and patients is configured as a strengthening of their involvement in safety promotion^(6)^. In this perspective, open disclosure is a strategy that aims to involve patients and professionals so that the latter recognize the mistakes made and discuss them with the parties involved, in order to strengthen PS and promote care improvement^(21)^.

Through CFA, the need to exclude FCP8 was also identified, which presented a factorial load well below the recommended one (0.36), and which had the following *wording: “No hospital onde fiz meu estágio, promovia-se uma cultura não punitiva, para que, caso ocorresse um erro, soubéssemos como evitar que ele se repetisse”.* PS culture is defined as an integrated pattern of individual and group values, attitudes, beliefs, skills and perceptions, aimed at minimizing harm to patients, and which determines commitment, style and proficiency with organizational safety management^(2)^. In this regard, PS culture, despite having broad communication as one of its characteristics^(6)^, involves a much broader context, which may have contributed to the item presenting a low factorial load (0.36) in the *“Franqueza na comunicação”* (FCP) dimension. Furthermore, it is important to consider that students inserted in clinical practice fields may be unaware of institutional processes related to the prevention of adverse events and error mitigation, which also justifies the result of low correlation of this item to the FCP dimension.

It is also worth mentioning CFH1, *“Nos serviços de saúde, em que realizei meus estágios, me explicaram as normas de segurança adotadas para os pacientes”,* which obtained a borderline factor loading (0.51). In the context of health care security, human factors are related to understanding the relationships between human beings and the environment in which it is involved^(6)^. The approach to human factors is important to improve care safety and non-application is strongly related to occurrence of errors and adverse events^(7)^. This result may indicate the need for an approach that aims to improve compliance with institutional processes as well as the dissemination of standards adopted to strengthen PS.

Regarding the LSPSQ structure, the division of its dimensions and the items that compose them, it is clear that, in general, they address aspects related to strengthening communication, patient involvement in safety, integration of human factors and systems, and improving quality of care and qualification of risk management. These aspects, together with teamwork, are recommended for the development of skills necessary for health professionals with a view to the adoption of safe practices^(6)^. Thus, it can be affirmed that the LSPSQ, Brazilian version^(12)^, reflects the theoretical construct to which it was proposed.

Discriminant validity also confirmed the LSPSQ, Brazilian version, structure^(12)^, as an item is expected to represent only the latent variable being verified^(18)^. The results of cross factor loadings showed that the items were properly allocated in each dimension.

With regard to adjustment indexes, in the original study, the LSPSQ^(14)^ author used only the GFI and the AGFI. However, in order to refine the analysis performed and allow different comparisons of adjustments, it is recommended to use other indexes concomitantly^(18)^. Among the most used are the CFI, TLI and RMSEA. The RMSEA is characterized as a residual-based index that assesses how much the proposed model differs from the real one, and the lower its value, the better the fit found^(18)^. On the other hand, the CFI and TLI are characterized as comparative indexes based on the use of an independence model (null model), and the higher their values, the better the result of adjustment^(18)^.

Another index used, χ^2^, assesses whether the proposed covariance matrix is equivalent to the sample matrix, and has the test static significance as an interpretation parameter, which must be non-significant However, because it is a sensitive measure to the sample size and because it tests a very rigid assumption, its individual use is not recommended^(18)^. As an alternative, it is possible to use the χ^2^/gl ratio assessment, which, for interpretation purposes, must have values less than three, which means a good fit of the^(19)^ model. In the present study, the fit indexes confirmed the degree to which the specified model reproduces the observed data, indicating instrument plausibility^(18)^.

The final model of the LSPSQ, Brazilian version, structure^(12)^, demonstrated by a path diagram, presents the correlation between the instrument dimensions. The FCP dimension showed a strong^(22)^, positive and significant (r=0.862; p < 0.001 ) correlation with the AP dimension. Communication with patients involves the sharing of knowledge between professionals and the possibility of discussing weaknesses in the environment that can cause safety failures^(6)^. The AP dimension incorporates in its items aspects related to risk management and the prevention of adverse events^(6)^. From this perspective, it was expected that these factors would be correlated, since both refer to the need to develop skills related to risk identification, application of knowledge for accident prevention and adoption of practices that promote a safe care environment.

The AP dimension showed a strong^(22)^, positive and significant correlation (r=0.687; p < 0.001) with the CFH dimension and, thus, as in the previous relationship, this correlation was expected. Human factors represent a strategic concept for PS, as they consider the interaction between humans and the environment, as well as the potential for care failure, in addition to encouraging the promotion of changes from the identification of occurrence of adverse events^(6-7)^. Thus, the development of these competencies is in line with improved safety and the prevention of errors and adverse events. Another correlation observed refers to the FCP dimension with CFH, which was also expected, since both represent the direction of safety knowledge and human behavior to promote safe care.

Other three significant correlations were observed; however, their magnitudes are considered weak^(22)^: AP and CE dimensions (r=0.179; p < 0.05); AP and CS dimensions (r=0.187; p < 0.05); and CFH and CS dimensions (r=0.264; p < 0.01). The CE dimension includes in its items aspects related to occurrence of situations favorable to errors and adverse events. For students, learning to identify these situations and act in order to prevent and mitigate their occurrences is an important competency to be developed for quality and safety of care^(7)^. The CS dimension, on the other hand, refers to the recognition of interaction between the parts of systems that represent health care, its complexity and its importance for prevention of errors and adverse events. Students must know this complexity and understand the influence of organizational structures and processes in conducting patient care in a systemic way as well as the importance of this approach for quality of care^(7)^.

An instruments’ reliability measures represent its ability to present consistent results and relate to how free the model is from measurement errors^(13)^. Internal consistency measure assesses the relationships between items and seeks to identify item response pattern accuracy^(13)^. In the original instrument, internal consistency was assessed using Cronbach’s alpha, composite reliability and item-total correlation^(14)^. Cronbach’s alpha use has been questioned, since it is characterized as an indicator that may underestimate reliability, and there are other measures for this assessment^(23)^. In the present study, we chose to use other measures of internal consistency in order to refine assessment and improve its interpretation. Adequate composite reliability measures in all dimensions and item-total correlation measures greater than 0.50 indicate that the LSPSQ, Brazilian version, is a reliable instrument.

### Study limitations

A limitation of this study is the number of respondents in the medical course, which was lower than expected for performing the multigroup CFA, despite being adequate for conducting CFA. As test validity is a process of investigation of evidence of validity, and because it is the first assessment of this instrument in the Brazilian population, it is suggested to conduct other studies that allow measurement assessment of invariance between groups, investigation of measurement errors and instrument responsiveness^(15)^. Another limitation refers to the absence of validity studies of LSPSQ in different cultural contexts, which makes it impossible to compare the validity evidence from Brazil with other populations.

On the other hand, one of the highlights of this study was in relation to sample diversity. We sought to include participants from different regions of Brazil, given the country’s territorial extension, as a way of covering different teaching contexts as well as possible existing cultural differences.

### Contributions to nursing, health, and public policies

The product made available in the present study will contribute to the advancement in knowledge and attitudes assessment in PS among undergraduate nursing and medicine students, due to the absence of validated instruments for this purpose in the Brazilian context. It is believed that the use of this instrument by educational institutions will allow the identification of training needs, and, in addition to the guidelines already proposed by the World Health Organization, it will be able to direct educational managers and teachers towards topic integration into the training curricula. The use of dynamic and interactive teaching tools, such as active pedagogical methodologies, is one of the ways to be developed in the teaching-learning process of students on PS, for encouraging critical reflection on behavior, attitude and problem solving in the face of challenges encountered during practical experience. In addition to this, it is believed that the application of LSPSQ may provide students with reflection on their knowledge, and thus reflect on improving quality of care.

## CONCLUSIONS

Assessing the LSPSQ, Brazilian version, psychometric properties showed adequate internal validity evidence and satisfactory reliability. It is, therefore, an appropriate instrument to measure knowledge and attitudes in PS among nursing and medical students in Brazil. It is expected that this instrument will contribute to the identification of knowledge gaps on the subject in order to highlight the importance of including PS in undergraduate health curricula. Greater attention to education on PS will impact, consequently, on qualification of care and on strengthening safety culture in health services.

## SUPPLEMENTARY MATERIAL

https://data.scielo.org/dataset.xhtml?persistentId=doi:10.48331/scielodata.TYNGTA

0034-7167-reben-76-02-e20210961-sup01
