# Supplementary material for: Psychometric properties of the Latino Students Patient Safety Questionnaire, Brazilian version
Source: Rev Bras Enferm. 2023 Feb 6;76(2):e20210961. doi: 10.1590/0034-7167-2021-0961 (PMC9901345; doi:10.1590/0034-7167-2021-0961)
Supplement: 0034-7167-reben-76-02-e20210961-sup01 [file 0034-7167-reben-76-02-e20210961-sup01.pdf]

N participante	Gênero	Idade anos	e estado de proce	enciatura o	ríodo você está cursando no m
001	Feminino	25	o Raimundo Nona	Enfermagem	9 período
002	Feminino	25	Floriano	Enfermagem	9 período
003	Feminino	26	rural de barao de	Enfermagem	9 período
004	Masculino	23	Parnarama	Enfermagem	9 período
005	Masculino	23	Condeúba Bahia	Enfermagem	9 período
006	Masculino	33	Picos PI	Enfermagem	9 período
007	Masculino	25	agem franca Mara	Enfermagem	9 período
008	Masculino	23	Raimundo Nonato	Enfermagem	9 período
009	Feminino	28	do Rio Branco Mir	Enfermagem	9 período
010	Masculino	31	Viçosa Minas Gerai	Medicina	10 período
011	Feminino	25	Teixeiras MG	Medicina	10 período
012	Feminino	24	nselheiro Lafaiete	Medicina	10 período
013	Masculino	26	Ubá MG	Enfermagem	9 período
014	Feminino	23	oeiro de Itapemiri	Enfermagem	9 período
015	Masculino	37	Passo Fundo RS	Medicina	11 período
016	Feminino	26	Ouro Branco MG	Enfermagem	9 período
017	Feminino	26	Passo Fundo RS	Medicina	11 período
018	Masculino	24	Fortaleza CE	Medicina	9 período
019	Feminino	23	Sobral Ceará	Medicina	10 período
020	Feminino	23	Floriano Piauí	Enfermagem	9 período
021	Feminino	21	PlanaltinaDF	Enfermagem	9 período
022	Feminino	22	Brasilia DF	Enfermagem	9 período
023	Feminino	24	Sobral Ceará	Medicina	9 período
024	Feminino	30	Floriano Piauí	Enfermagem	9 período
025	Feminino	22	strito Federal Gan	Enfermagem	9 período
026	Feminino	23	Petrolina PE	Medicina	11 período
027	Masculino	22	Petrolina PE	Medicina	10 período
028	Feminino	23	Ituiutaba MG	Medicina	10 período
029	Feminino	24	itu sp	Medicina	10 período
030	Feminino	27	Belo jardim	Medicina	10 período
031	Feminino	28	tória da conquist	Enfermagem	9 período
032	Feminino	24	amirim e Pernamb	Medicina	9 período
033	Feminino	22	Petrolina PE	Medicina	10 período
034	Feminino	22	Fortaleza CE	Enfermagem	9 período
035	Feminino	24	Fortaleza	Enfermagem	9 período
036	Feminino	26	Fortaleza Ceará	Enfermagem	9 período
037	Masculino	29	Juazeiro Bahia	Medicina	10 período
038	Feminino	21	uatinga Distrito Fe	Enfermagem	9 período
039	Feminino	31	Fortaleza CE	Enfermagem	10 período
040	Masculino	27	esterro do melo M	Enfermagem	9 período
041	Feminino	24	Carmo da Mata MG	Enfermagem	8 período
042	Feminino	27	ESPACHO MINAS	Enfermagem	9 período
043	Feminino	28	Passo Fundo	Medicina	10 período
044	Feminino	21	Divinópolis	Enfermagem	9 período
045	Masculino	24	Passo Fundo RS	Medicina	12 período
046	Feminino	34	FORTALEZA CEARÁ	Enfermagem	9 período
047	Feminino	22	Fortaleza Ceará	Enfermagem	9 período
048	Feminino	24	Fortaleza CEARA	Enfermagem	10 período
049	Feminino	23	Fortaleza CE	Enfermagem	10 período
050	Feminino	23	Belo Horizonte	Enfermagem	8 período
051	Feminino	24	Fortaleza CE	Enfermagem	9 período
052	Feminino	25	Juazeiro Bahia	Medicina	9 período
053	Feminino	23	Brasília	Enfermagem	9 período
054	Feminino	22	ândia Distrito Fed	Enfermagem	9 período
055	Masculino	24	Fortaleza CE	Enfermagem	9 período
056	Masculino	24	Belo Horizonte MG	Medicina	11 período
057	Feminino	24	Fortaleza Ceará	Enfermagem	9 período
058	Feminino	24	DIVINÓPOLIS	Enfermagem	9 período

059	Feminino	28	inópolis Minas Ge	Enfermagem	9 período
060	Masculino	24	raraquara São Pau	Enfermagem	8 período
061	Feminino	30	Divinópolis MG	Enfermagem	8 período
062	Feminino	29	Divinopolis	Enfermagem	9 período
063	Masculino	23	DIVINOPOLIS	Enfermagem	8 período
064	Feminino	23	Fortaleza CE	Enfermagem	10 período
065	Feminino	21	Samambaia DF	Enfermagem	9 período
066	Masculino	30	Teutônia RS	Medicina	12 período
067	Masculino	29	Petrolin PE	Medicina	11 período
068	Feminino	24	Divinópolis	Enfermagem	9 período
069	Masculino	22	Divinópolis MG	Enfermagem	8 período
070	Feminino	25	ntagem Minas Ger	Enfermagem	9 período
071	Feminino	24	Brasília DF	Enfermagem	9 período
072	Feminino	28	Vicosa MG	Enfermagem	9 período
073	Feminino	23	FORTALEZA	Enfermagem	9 período
074	Masculino	26	Passo Fundo RS	Medicina	10 período
075	Feminino	21	Fortaleza Ceará	Enfermagem	9 período
076	Feminino	24	inópolis Minas Ge	Enfermagem	9 período
077	Feminino	22	ambuí Minas Gera	Enfermagem	9 período
078	Masculino	21	Brumadinho MG	Enfermagem	9 período
079	Feminino	22	Belo Horizonte MG	Enfermagem	9 período
080	Feminino	23	Horizonte Minas G	Enfermagem	9 período
081	Masculino	24	Horizonte Minas G	Enfermagem	9 período
082	Masculino	28	Leopoldo Minas G	Enfermagem	9 período
083	Feminino	24	Belo Horizonte	Enfermagem	9 período
084	Feminino	31	Curitiba Paraná	Enfermagem	9 período
085	Feminino	22	Leopoldo Minas G	Enfermagem	9 período
086	Feminino	33	Viçosa MG	Enfermagem	9 período
087	Feminino	23	Viçosa MG	Enfermagem	9 período
088	Masculino	26	Nata RN	Medicina	12 período
089	Feminino	22	Sobral CE	Medicina	10 período
090	Feminino	25	Belo Horizonte MG	Enfermagem	9 período
091	Feminino	23	Divinópolis MG	Enfermagem	9 período
092	Feminino	24	tabira Minas Gerai	Medicina	11 período
093	Feminino	24	Perdões MG	Medicina	11 período
094	Masculino	34	Horizonte Minas G	Medicina	11 período
095	Feminino	24	Cruzeiro SP	Medicina	10 período
096	Masculino	23	va Lima Minas Ger	Medicina	11 período
097	Masculino	22	taúna Minas Gerai	Medicina	9 período
098	Feminino	26	Belo Horizonte MG	Medicina	10 período
099	Masculino	23	inopolis Minas Ge	Medicina	10 período
100	Feminino	23	Contagem MG	Medicina	10 período
101	Masculino	27	omandel Minas Ge	Medicina	11 período
102	Masculino	26	nópolis Minas Ger	Medicina	11 período
103	Feminino	22	Betim MG	Medicina	9 período
104	Feminino	24	de Minas Minas G	Medicina	9 período
105	Feminino	24	Divinópolis	Medicina	10 período
106	Feminino	23	Antônio do Mont	Medicina	10 período
107	Feminino	24	Cláudio MG	Enfermagem	9 período
108	Feminino	24	Divinópolis MG	Enfermagem	9 período
109	Masculino	31	Fortaleza	Enfermagem	10 período
110	Feminino	23	Horizonte Ceará	Enfermagem	10 período
111	Masculino	22	Picos Piauí	Enfermagem	9 período
112	Masculino	27	Tauá Ceará	Enfermagem	9 período
113	Feminino	23	Picos PIAUÍ	Enfermagem	9 período
114	Feminino	22	Picos Pi	Enfermagem	9 período
115	Masculino	22	MPOS SALES CEA	Enfermagem	9 período
116	Masculino	22	Picos PI	Enfermagem	9 período
117	Feminino	29	Divinópolis	Medicina	11 período

118	Feminino	24	ıpo Belo Minas Ge	Medicina	11 período
119	Feminino	22	Picos Piauí	Enfermagem	9 período
120	Feminino	22	Miguel do Tapuio	Enfermagem	9 período
121	Feminino	26	Horizonte Minas (	Medicina	10 período
122	Masculino	22	Picos	Enfermagem	9 período
123	Masculino	23	Fortaleza ce	Enfermagem	10 período
124	Masculino	24	Picos PI	Enfermagem	9 período
125	Masculino	21	Uberaba MG	Medicina	9 período
126	Feminino	28	Itaúna	Medicina	12 período
127	Masculino	22	Oliveira MG	Medicina	9 período
128	Feminino	23	Fortaleza Ceará	Enfermagem	10 período
129	Feminino	23	Divinópolis MG	Enfermagem	8 período
130	Feminino	22	Taguatinga DF	Enfermagem	9 período
131	Feminino	25	Divinópolis MG	Medicina	10 período
132	Masculino	33	PETROLINA PE	Enfermagem	9 período
133	Feminino	23	Fortaleza	Enfermagem	10 período
134	Feminino	27	Filadélfia Ba	Medicina	10 período
135	Feminino	34	JUAZEIRO BA	Enfermagem	10 período
136	Feminino	23	Rio Acima MG	Enfermagem	10 período
137	Feminino	23	Belo Horizonte MG	Enfermagem	10 período
138	Feminino	24	Viçosa Minas Gerai	Enfermagem	9 período
139	Masculino	24	Sobral CE	Medicina	10 período
140	Masculino	26	Fortaleza CE	Medicina	11 período
141	Feminino	24	Fortaleza Ceará	Enfermagem	10 período
142	Feminino	27	divinopolis	Medicina	11 período
143	Feminino	26	JANAÚBA MG	Medicina	11 período
144	Feminino	24	São Paulo SP	Medicina	11 período
145	Feminino	25	Pitangui MG	Enfermagem	9 período
146	Masculino	22	Picos PI	Medicina	9 período
147	Masculino	26	Belo Horizonte MG	Enfermagem	10 período
148	Feminino	24	Belo Horizonte MG	Enfermagem	10 período
149	Feminino	22	Bh mg	Enfermagem	10 período
150	Feminino	25	Sobral ceará	Medicina	11 período
151	Feminino	22	picos piauí	Enfermagem	9 período
152	Feminino	21	Francisco do Mara	Enfermagem	10 período
153	Feminino	26	divinópolis	Medicina	10 período
154	Feminino	29	Divinópolis	Medicina	11 período
155	Feminino	22	Picos Pi	Enfermagem	9 período
156	Feminino	25	Pitangui mg	Enfermagem	9 período
157	Masculino	24	ião Peixoto São Pa	Enfermagem	8 período
158	Feminino	26	Belo Horizonte MG	Enfermagem	9 período
159	Feminino	22	inópolis Minas Ge	Enfermagem	9 período
160	Feminino	23	ta Luzia Minas Ge	Enfermagem	9 período
161	Masculino	23	Picos Piauí	Enfermagem	9 período
162	Masculino	28	esterro do melo M	Enfermagem	9 período
163	Feminino	24	nópolis Minas Ge	Enfermagem	8 período
164	Feminino	24	ronel Fabriciano M	Medicina	9 período
165	Masculino	24	bacena Minas Gei	Medicina	9 período
166	Masculino	23	Lagoa da Prata MG	Medicina	9 período
167	Masculino	24	Abre Campo MG	Medicina	9 período
168	Masculino	32	Viçosa Minas Gerai	Medicina	9 período
169	Feminino	21	Chapecó SC	Enfermagem	9 período
170	Feminino	21	apecó Santa Catar	Enfermagem	9 período
171	Feminino	23	có Santa Catarina,	Enfermagem	9 período
172	Feminino	22	Cotegipe Rio Gran	Enfermagem	9 período
173	Masculino	24	Raimundo Nonato	Enfermagem	10 período
174	Feminino	22	Chapecó SC	Enfermagem	9 período
175	Masculino	24	Belo Horizonte MG	Enfermagem	8 período
176	Masculino	23	Divinópolis MG	Enfermagem	8 período

177	Masculino	29	Divinópolis MG	Medicina	9 período
178	Feminino	22	Divinópolis MG	Medicina	10 período
179	Feminino	50	3elo Horizonte MC	Enfermagem	9 período
180	Feminino	24	BH MG	Enfermagem	10 período
181	Feminino	35	Itabira MG	Medicina	12 período
182	Feminino	23	ratinga Minas Ger	Medicina	9 período
183	Masculino	25	Fortaleza Ceará	Medicina	12 período
184	Feminino	24	Petrolina PE	Enfermagem	9 período
185	Feminino	27	Passo Fundo RS	Medicina	11 período
186	Feminino	22	Viçosa MG	Medicina	10 período
187	Feminino	28	Sete Lagoas MG	Enfermagem	10 período
188	Feminino	24	3elo Horizonte MC	Enfermagem	9 período
189	Feminino	27	Passo Fundo RS	Medicina	12 período
190	Feminino	32	Curitiba PR	Enfermagem	9 período
191	Feminino	25	r do Bonfim Bahi	Medicina	9 período
192	Feminino	29	Viçosa Mg	Enfermagem	9 período
193	Feminino	27	Fortaleza Ceará	Enfermagem	10 período
194	Masculino	22	São Paulo SP	Enfermagem	8 período
195	Feminino	23	Divinópolis MG	Enfermagem	8 período
196	Feminino	24	Uberaba MG	Medicina	9 período
197	Feminino	24	Lavras MG	Medicina	12 período
198	Feminino	24	divinopolis MG	Medicina	9 período
199	Feminino	33	'içosa Minas Gerai	Medicina	9 período
200	Masculino	34	Belo Horizonte mg	Medicina	12 período
201	Masculino	22	Uberaba MG	Medicina	9 período
202	Feminino	26	Horizonte Minas G	Medicina	10 período
203	Feminino	27	BH	Medicina	10 período
204	Masculino	23	Divinópolis MG	Medicina	9 período
205	Masculino	25	Divinópolis MG	Medicina	9 período
206	Feminino	26	Teresina	Enfermagem	10 período
207	Feminino	23	Fortaleza	Enfermagem	10 período
208	Feminino	25	Fortaleza	Enfermagem	10 período
209	Feminino	23	Domingos do Prata	Enfermagem	9 período
210	Feminino	23	Brasília DF	Enfermagem	9 período
211	Masculino	24	BH	Enfermagem	10 período
212	Feminino	37	oão Pessoa Paraib	Enfermagem	10 período
213	Feminino	27	abirito Minas Gera	Enfermagem	10 período
214	Feminino	30	3elo Horizonte MC	Enfermagem	9 período
215	Feminino	23	BELO HORIZONTE	Enfermagem	9 período
216	Feminino	25	Fortaleza Ceará	Enfermagem	10 período
217	Feminino	23	Bento do Sapucaí	Enfermagem	8 período
218	Feminino	21	Fortaleza CE	Enfermagem	10 período

Universidade onde realiza os seus estudos	região do brasil	de estágio
UFPI	Nordeste	Sim
UFPI	Nordeste	Sim
UFPI	Nordeste	Sim
UFPI	Nordeste	Sim
UFC	Nordeste	Sim
UFPI	Nordeste	Sim
Ufpi	Nordeste	Sim
UFPI	Nordeste	Sim
UFV	Sudeste	Sim
UFV	Sudeste	Sim
UFV	Sudeste	Sim
UFV	Sudeste	Sim
UFV	Sudeste	Sim
UFV	Sudeste	Sim
UFFS	Sul	Sim
UFV	Sudeste	Sim
UFFS	Sul	Sim
UFC	Nordeste	Sim
UFC	Nordeste	Sim
UFPI	Nordeste	Sim
UNB	Centro-Oeste	Sim
UNB	Centro-Oeste	Sim
UFC	Nordeste	Sim
UFPI	Nordeste	Sim
UNB	Centro-Oeste	Sim
Univasf	Nordeste	Sim
UNIVASF	Nordeste	Sim
UNIVASF	Nordeste	Sim
univasf	Nordeste	Sim
UNIVASF	Nordeste	Sim
UFPI	Nordeste	Sim
Univasf	Nordeste	Sim
UNIVASF	Nordeste	Sim
UFC	Nordeste	Sim
UFC	Nordeste	Sim
UFC	Nordeste	Sim
UNIVASF	Nordeste	Sim
UNB	Centro-Oeste	Sim
UFC	Nordeste	Sim
UFSJ	Sudeste	Sim
UFSJ	Sudeste	Sim
UFSJ	Sudeste	Sim
UFFS	Sul	Sim
UFSJ	Sudeste	Sim
uffs	Sul	Sim
UFC	Nordeste	Sim
UFC	Nordeste	Sim
UFC	Nordeste	Sim
UFC	Nordeste	Sim
UFSJ	Sudeste	Sim
UFC	Nordeste	Sim
Univasf	Nordeste	Sim
UNB	Centro-Oeste	Sim
UNB	Centro-Oeste	Sim
UFC	Nordeste	Sim
UNIVASF	Nordeste	Sim
UFC	Nordeste	Sim
UFSJ	Sudeste	Sim

UFSJ	Sudeste	Sim
UFSJ	Sudeste	Sim
UFSJ	Sudeste	Sim
UFSJ	Sudeste	Sim
UFSJ	Sudeste	Sim
UFC	Nordeste	Sim
UnB	Centro-Oeste	Sim
UNIVASF	Nordeste	Sim
UNIVASF	Nordeste	Sim
UFSJ	Sudeste	Sim
UFSJ	Sudeste	Sim
UFSJ	Sudeste	Sim
UNB	Centro-Oeste	Sim
UFV	Sudeste	Sim
UFC	Nordeste	Sim
UFFS	Sul	Sim
UFC	Nordeste	Sim
UFSJ	Sudeste	Sim
UFSJ	Sudeste	Sim
UFMG	Sudeste	Sim
UFMG	Sudeste	Sim
UFMG	Sudeste	Sim
UFMG	Sudeste	Sim
UFMG	Sudeste	Sim
UFMG	Sudeste	Sim
UFMG	Sudeste	Sim
UFMG	Sudeste	Sim
UFV	Sudeste	Sim
UFV	Sudeste	Sim
UFC	Nordeste	Sim
UFC	Nordeste	Sim
UFMG	Sudeste	Sim
UFSJ	Sudeste	Sim
UFSJ	Sudeste	Sim
UFSJ	Sudeste	Sim
UFSJ	Sudeste	Sim
UFSJ	Sudeste	Sim
UFSJ	Sudeste	Sim
UFSJ	Sudeste	Sim
UFSJ	Sudeste	Sim
UFSJ	Sudeste	Sim
UFSJ	Sudeste	Sim
UFSJ	Sudeste	Sim
UFSJ	Sudeste	Sim
UFSJ	Sudeste	Sim
UFSJ	Sudeste	Sim
UFSJ	Sudeste	Sim
UFSJ	Sudeste	Sim
UFSJ	Sudeste	Sim
UFSJ	Sudeste	Sim
UFC	Nordeste	Sim
UFC	Nordeste	Sim
UFPI	Nordeste	Sim
UFPI	Nordeste	Sim
UFPI	Nordeste	Sim
UFPI	Nordeste	Sim
UFPI	Nordeste	Sim
UFPI	Nordeste	Sim
UFSJ	Sudeste	Sim

UFSJ	Sudeste	Sim
UFPI	Nordeste	Sim
UFPI	Nordeste	Sim
UFSJ	Sudeste	Sim
UFPI	Nordeste	Sim
UFC	Nordeste	Sim
UFPI	Nordeste	Sim
UFSJ	Sudeste	Sim
Ufsj	Sudeste	Sim
UFSJ	Sudeste	Sim
UFC	Nordeste	Sim
UFSJ	Sudeste	Sim
UNB	Centro-Oeste	Sim
UFSJ	Sudeste	Sim
UNIVASF	Nordeste	Sim
UFC	Nordeste	Sim
UNIVASF	Nordeste	Sim
UNIVASF	Nordeste	Sim
UFMG	Sudeste	Sim
UFMG	Sudeste	Sim
UFV	Sudeste	Sim
UFC	Nordeste	Sim
UFC	Nordeste	Sim
UFC	Nordeste	Sim
UFSJ	Sudeste	Sim
UFSJ	Sudeste	Sim
UFSJ	Sudeste	Sim
Ufsj	Sudeste	Sim
UNIVASF	Nordeste	Sim
UFMG	Sudeste	Sim
UFMG	Sudeste	Sim
Ufmg	Sudeste	Sim
Ufc	Nordeste	Sim
UFPI	Nordeste	Sim
UFPI	Nordeste	Sim
UFSJ	Sudeste	Sim
UFSJ	Sudeste	Sim
UFPI	Nordeste	Sim
Ufsj	Sudeste	Sim
UFSJ	Sudeste	Sim
UFMG	Sudeste	Sim
UFSJ	Sudeste	Sim
UFMG	Sudeste	Sim
UFPI	Nordeste	Sim
UFSJ	Sudeste	Sim
UFSJ	Sudeste	Sim
UFV	Sudeste	Sim
UFV	Sudeste	Sim
UFV	Sudeste	Sim
UFV	Sudeste	Sim
UFV	Sudeste	Sim
UFFS	Sul	Sim
UFFS	Sul	Sim
UFFS	Sul	Sim
UFFS	Sul	Sim
UFPI	Nordeste	Sim
UFFS	Sul	Sim
UFSJ	Sudeste	Sim
UFSJ	Sudeste	Sim

UFSJ	Sudeste	Sim
UFSJ	Sudeste	Sim
UFMG	Sudeste	Sim
UFMG	Sudeste	Sim
UFV	Sudeste	Sim
UFV	Sudeste	Sim
UFC	Sudeste	Sim
UNIVASF	Nordeste	Sim
UFFS	Sul	Sim
UFV	Sudeste	Sim
UFMG	Sudeste	Sim
UFMG	Sudeste	Sim
UFFS	Sul	Sim
UFMG	Sudeste	Sim
UNIVASF	Nordeste	Sim
UFV	Sudeste	Sim
UFC	Nordeste	Sim
UFSJ	Sudeste	Sim
UFSJ	Sudeste	Sim
UFSJ	Sudeste	Sim
UFSJ	Sudeste	Sim
UFSJ	Sudeste	Sim
UFSJ	Sudeste	Sim
UFSJ	Sudeste	Sim
UFSJ	Sudeste	Sim
UFSJ	Sudeste	Sim
UFSJ	Sudeste	Sim
UFSJ	Sudeste	Sim
UFSJ	Sudeste	Sim
UFPI	Nordeste	Sim
UFC	Nordeste	Sim
UFC	Nordeste	Sim
UFV	Sudeste	Sim
UNB	Centro-Oeste	Sim
UFMG	Sudeste	Sim
UNIVASF	Nordeste	Sim
UFV	Sudeste	Sim
UFMG	Sudeste	Sim
UFMG	Sudeste	Sim
UFC	Nordeste	Sim
UFSJ	Sudeste	Sim
UFC	Nordeste	Sim

e leitos existentes no hospital onde r	po estágio a	rvisor de es	mação na ár	Sim. Qual?	algum serv
150	enção Primá	Sim	Não		Não
40	enção Primá	Sim	Não		Não
Faco ideia, mas é mais de 200 com ce	Hospitalar	Não	Não		Não
1000	Hospitalar	Em partes	Não		Não
110	enção Primá	Sim	Não		Não
Não sei	enção Primá	Sim	Não		Não
100	enção Primá	Sim	Não		Não
150	Hospitalar	Em partes	Não		Não
100	Hospitalar	Sim	Não		Não
50	Outro	Sim	Não		Não
50	enção Primá	Sim	Não		Não
120	Hospitalar	Sim	Não		Não
130	Hospitalar	Não	Não		Não
100	enção Primá	Sim	Não		Não
200	Hospitalar	Sim	Sim	Psicologia	Não
0	enção Primá	Sim	Não		Não
800	Hospitalar	Em partes	Não		Não
600	Hospitalar	Em partes	Não		Não
15	Hospitalar	Não	Não		Não
198	Hospitalar	Em partes	Não		Não
não sei infomar	enção Primá	Sim	Não		Não
Hospital de grande porte	enção Primá	Sim	Sim	Tecnico em enfermagem	Não
25	Hospitalar	Sim	Não		Não
200	Hospitalar	Sim	Sim	Técnico em vigilância em saúde	Não
150	enção Primá	Sim	Não		Não
100	enção Primá	Sim	Não		Não
130	Hospitalar	Sim	Não		Não
150	Hospitalar	Sim	Não		Não
100	Hospitalar	Sim	Não		Não
50	Hospitalar	Sim	Não		Não
Não sei informar	Hospitalar	Sim	Não		Não
Não sei ao certo	Hospitalar	Sim	Não		Não
130	Hospitalar	Sim	Não		Não
50	Hospitalar	Em partes	Não		Não
400	enção Primá	Em partes	Não		Não
70	Hospitalar	Sim	Não		Não
0	enção Primá	Sim	Não		Não
150	enção Primá	Sim	Não		Não
262	enção Primá	Sim	Não		Não
10	enção Primá	Em partes	Não		Não
Não tenho conhecimento	Hospitalar	Em partes	Não		Não
SEIS	enção Primá	Sim	Não		Não
800	Hospitalar	Em partes	Não		Não
Aproximadamente 380	enção Primá	Em partes	Não		Não
900	Hospitalar	Sim	Não		Não
48	enção Primá	Sim	Não		Não
198	Hospitalar	Sim	Sim	Técnico em Enfermagem	Não
243	enção Primá	Sim	Não		Não
541	enção Primá	Em partes	Sim	técnico de enfermagem	Sim
40	Hospitalar	Sim	Não		Não
200	Hospitalar	Não	Não		Não
400	Hospitalar	Sim	Não		Não
294	enção Primá	Não	Não		Não
275	enção Primá	Sim	Não		Não
260	Hospitalar	Sim	Não		Não
100	Hospitalar	Em partes	Não		Não
541	Hospitalar	Em partes	Não	Técnico em Enfermagem	Não
40	enção Primá	Sim	Não		Não

Não faço ideia	enção Primá	Sim	Não		Não
35	Hospitalar	Em partes	Não		Não
informar, mas no meu setor existe 3	Hospitalar	Em partes	Sim	Téc. enfemagem.	Sim
90	enção Primá	Sim	Não		Não
(de acordo com uma reportagem do	Hospitalar	Sim	Não		Não
243	enção Primá	Em partes	Não		Não
300	enção Primá	Sim	Não		Não
100	Hospitalar	Sim	Não		Não
40	Hospitalar	Em partes	Não		Não
42 no setor	enção Primá	Sim	Não		Não
20	Outro	Sim	Não		Não
48	enção Primá	Sim	Não		Não
Não sei	enção Primá	Em partes	Não		Não
0	enção Primá	Sim	Não		Não
198	Hospitalar	Em partes	Não		Não
2000	enção Primá	Sim	Não		Não
Não sei dizer com exatidão	Hospitalar	Em partes	Não		Não
50	enção Primá	Em partes	Não		Não
ão sei. Mas no setor que fiz estágio l	enção Primá	Sim	Não		Não
504	enção Primá	Em partes	Não		Não
504	enção Primá	Não	Não		Não
Não fiz o estágio hospitalar	enção Primá	Sim	Não		Não
Não sei informar	enção Primá	Em partes	Não		Não
-	enção Primá	Não	Não		Não
630	enção Primá	Não	Não		Não
Não	enção Primá	Sim	Sim	Medicina Veterinária	Não
2400	enção Primá	Em partes	Não		Não
Não sei informar	Hospitalar	Sim	Não		Não
20	Hospitalar	Em partes	Não		Não
400	Hospitalar	Sim	Não		Não
500	Hospitalar	Sim	Não		Não
Não sei	enção Primá	Sim	Não		Não
400	enção Primá	Sim	Não		Não
400	Hospitalar	Sim	Sim	Técnico em enfermagem	Não
Não sei dizer	Hospitalar	Sim	Não		Não
300	Hospitalar	Em partes	Não		Não
300	Hospitalar	Sim	Não		Não
350	Hospitalar	Em partes	Não		Não
300	Hospitalar	Sim	Não		Não
100	Hospitalar	Sim	Não		Não
200	Hospitalar	Sim	Não		Não
400	Hospitalar	Sim	Não		Não
39 leitos de UTI	Hospitalar	Sim	Não		Não
300	Hospitalar	Sim	Não		Não
300	Hospitalar	Sim	Não		Não
que 400, não achei a informação na	Hospitalar	Sim	Não		Não
300	Hospitalar	Sim	Não		Não
NÃO SEI	Outro	Sim	Não		Não
300	enção Primá	Sim	Não		Não
100	enção Primá	Sim	Não		Não
6000	enção Primá	Sim	Não		Não
Não sei responder	enção Primá	Sim	Sim	Técnico de enfermagem	Não
8	Hospitalar	Sim	Não		Não
aproximadamente 150 leitos	Hospitalar	Sim	Sim	Técnico em Enfermagem	Não
100	Hospitalar	Sim	Não		Não
30	Hospitalar	Sim	Não		Não
150	Hospitalar	Sim	Não		Não
100	Hospitalar	Em partes	Não		Não
600	Hospitalar	Sim	Não		Não

300	Hospitalar	Em partes	Não		Não
Mais de 100, não sei afirmar.	Hospitalar	Sim	Não		Não
35	Hospitalar	Sim	Não		Não
350	Hospitalar	Sim	Não		Não
10	Hospitalar	Sim	Sim	Técnico de enfermagem	Não
262	enção Primá	Sim	Não		Não
50	Hospitalar	Em partes	Sim	Técnico em Enfermagem	Sim
0	enção Primá	Sim	Não		Não
100	Hospitalar	Sim	Não		Não
300	Hospitalar	Sim	Não		Não
300	enção Primá	Sim	Não		Não
20	Hospitalar	Sim	Não		Não
30	enção Primá	Em partes	Não		Não
80	Hospitalar	Sim	Não		Não
135	enção Primá	Sim	Sim	TEC ENFERMAGEM	Sim
40	enção Primá	Em partes	Não		Não
500	Hospitalar	Sim	Não		Não
20	enção Primá	Sim	Não		Não
30	Hospitalar	Sim	Sim	Técnico em Enfermagem	Não
13	Hospitalar	Sim	Não		Não
Não sei ao certo	enção Primá	Sim	Não		Não
150	Hospitalar	Sim	Não		Não
150	Hospitalar	Sim	Não		Não
262	enção Primá	Em partes	Não		Não
São João de Deus não sei o número	Hospitalar	Sim	Não		Não
352	Hospitalar	Sim	Não		Não
Não sei	Hospitalar	Sim	Não		Não
230	enção Primá	Em partes	Não		Não
255	Hospitalar	Sim	Não		Não
500	Hospitalar	Sim	Não		Não
600	Hospitalar	Sim	Não		Não
400	Hospitalar	Sim	Não		Sim
400	Hospitalar	Sim	Não		Não
50	Hospitalar	Em partes	Não		Não
200	Hospitalar	Em partes	Não		Não
80	Hospitalar	Em partes	Não		Não
600	Hospitalar	Sim	Não		Não
30	Hospitalar	Sim	Sim	Agente comunitario de saude	Não
35	enção Primá	Em partes	Não		Não
muitos, não sei quantos	Hospitalar	Em partes	Não		Não
250	enção Primá	Sim	Não		Sim
Mais que 326	enção Primá	Em partes	Não		Não
não sei	enção Primá	Sim	Não		Não
40	Hospitalar	Sim	Não		Não
10	enção Primá	Sim	Não		Não
300	Hospitalar	Sim	Não		Não
Não tenho ideia.	Hospitalar	Em partes	Não		Não
Não sei informar	Outro	Sim	Não		Não
15	Hospitalar	Sim	Não		Não
Nao sei	enção Primá	Sim	Sim		Sim
100	Outro	Em partes	Não		Não
	Atenção Primá	Sim	Não		Não
250	enção Primá	Sim	Não		Não
100	enção Primá	Em partes	Não		Não
Não sei	Hospitalar	Sim	Não		Não
300	enção Primá	Sim	Não		Não
293	Hospitalar	Em partes	Não		Não
50	Hospitalar	Sim	Não		Não
50	Hospitalar	Sim	Não		Não

200	ençãо Primá	Sim	Sim	Educação Física	Sim
300	Hospitalar	Sim	Não		Não
Seis	ençãо Primá	Sim	Sim	Psicologia	Não
16	Hospitalar	Sim	Sim		Não
60	Hospitalar	Em partes	Sim	Fonoaudiologia	Não
60	Hospitalar	Em partes	Não		Não
450	Hospitalar	Sim	Não		Sim
.	ençãо Primá	Sim	Não		Não
600	Hospitalar	Em partes	Não		Não
Não sei	ençãо Primá	Sim	Sim		Não
504	Hospitalar	Sim	Não		Não
Hospital das clínicas UFMG	ençãо Primá	Sim	Não		Não
aproximadamente 100	Hospitalar	Sim	Não		Não
não sei informar	ençãо Primá	Sim	Sim	Medicina Veterinaria	Não
200	Hospitalar	Em partes	Não		Não
106	ençãо Primá	Em partes	Não		Não
20	ençãо Primá	Em partes	Não		Sim
200	Hospitalar	Em partes	Não		Não
não sei infomar	Outro	Em partes	Não		Sim
Não sei	Outro	Sim	Sim		Sim
350	ençãо Primá	Em partes	Não		Não
-	ençãо Primá	Sim	Não		Não
-	ençãо Primá	Sim	Sim	Enfermagem	Sim
400	ençãо Primá	Em partes	Não		Não
10	ençãо Primá	Sim	Não		Não
300	Hospitalar	Sim	Não		Não
300	Hospitalar	Sim	Não		Não
300	ençãо Primá	Sim	Não		Não
X	ençãо Primá	Sim	Não		Não
150	ençãо Primá	Em partes	Sim	Técnico de enfermagem	Sim
21	ençãо Primá	Sim	Não		Não
30	ençãо Primá	Em partes	Não		Não
Não sei	ençãо Primá	Em partes	Sim	Técnico em enfermagem	Não
40	ençãо Primá	Em partes	Não		Não
10	Hospitalar	Em partes	Não		Não
NÃO SEI INFORMAR	Hospitalar	Sim	Não		Não
27	ençãо Primá	Sim	Não		Não
30	ençãо Primá	Em partes	Não		Não
537	ençãо Primá	Em partes	Não		Não
500	Outro	Em partes	Não		Não
Não sei	ençãо Primá	Sim	Não		Não
541	Outro	Sim	Não		Não

Sim. Qual?	que frequên	FCP1	FCP2	FCP3	FCP4	FCP5	FCP6	FCP7	FCP8
	Sempre	3	5	4	5	5	4	5	4
	Sempre	4	5	4	5	4	4	5	5
	Sempre	4	4	5	4	4	4	4	4
	Poucas veze:	4	3	3	3	2	2	3	2
	Sempre	5	5	5	5	5	5	5	3
	Poucas veze:	4	4	3	4	3	2	4	3
	Poucas veze:	4	4	3	4	4	4	4	4
	Poucas veze:	2	4	2	4	4	2	5	3
	Sempre	3	3	3	3	5	2	5	2
	Poucas veze:	4	4	3	2	2	2	5	4
	Sempre	4	4	4	4	4	2	4	4
	Poucas veze:	3	5	4	5	4	4	5	3
	Sempre	3	4	3	3	3	2	4	3
	Sempre	2	3	4	3	2	4	4	3
	Poucas veze:	3	5	4	4	4	4	5	4
	Sempre	4	4	4	4	4	3	4	2
	Poucas veze:	3	2	3	4	2	4	4	4
	Sempre	4	4	4	5	4	4	5	5
	Nunca	1	1	1	1	1	3	4	3
	Sempre	2	4	3	4	4	3	4	2
	Sempre	2	4	2	4	2	3	4	3
	Poucas veze:	1	3	1	3	3	3	4	3
	Sempre	2	4	3	1	2	4	4	3
	Sempre	3	5	4	4	4	4	4	1
	Sempre	4	4	4	4	5	5	5	2
	Poucas veze:	4	4	3	2	3	4	5	3
	Sempre	4	4	4	5	5	4	5	5
	Sempre	1	4	3	5	2	2	5	2
	Poucas veze:	1	4	4	3	4	2	4	3
	Sempre	2	4	4	4	3	3	4	2
	Sempre	2	4	4	5	4	2	4	1
	Poucas veze:	3	5	4	5	3	4	4	3
	Sempre	4	4	5	3	3	2	4	2
	Sempre	3	5	4	4	5	3	5	5
	Sempre	1	4	4	4	3	2	4	3
	Sempre	2	5	1	4	2	1	3	4
	Sempre	3	4	5	5	3	3	4	3
	Sempre	4	4	4	5	3	4	4	4
	Sempre	3	4	4	3	3	4	5	4
	Poucas veze:	3	4	4	5	4	3	3	4
	Sempre	3	4	4	4	3	3	4	2
	Sempre	4	5	4	5	5	4	5	3
	Poucas veze:	4	3	3	3	2	3	4	3
	Sempre	3	5	3	5	5	5	5	1
	Poucas veze:	4	2	4	4	3	4	4	3
	Sempre	2	4	4	4	4	4	4	4
	Sempre	4	5	4	4	4	5	4	4
	Sempre	2	4	2	4	4	2	4	4
no técnica c	Sempre	5	5	5	3	5	5	5	4
	Sempre	1	5	2	4	1	4	5	1
	Sempre	2	4	2	3	4	3	4	4
	Poucas veze:	3	4	4	4	3	4	4	2
	Sempre	3	4	4	4	2	3	4	2
	Sempre	4	5	5	5	4	3	5	3
	Sempre	3	5	4	4	4	4	5	3
	Poucas veze:	4	4	3	3	4	4	5	4
	Sempre	4	4	5	4	4	3	5	2
	Sempre	4	5	5	4	4	4	5	3

	Poucas vezes	4	4	5	5	4	5	5	1
	Poucas vezes	5	4	4	4	4	4	5	4
sericórdia d	Sempre	3	5	3	5	3	2	5	2
	Poucas vezes	2	2	2	2	2	2	4	2
	Sempre	4	5	5	5	5	5	5	3
	Poucas vezes	2	4	3	1	2	1	5	3
	Sempre	2	4	4	3	4	2	4	2
	Sempre	5	5	4	5	4	4	4	3
	Sempre	3	5	3	4	4	4	4	5
	Poucas vezes	4	4	5	5	5	4	5	4
	Sempre	3	5	4	4	4	3	4	1
	Sempre	2	4	4	2	4	2	4	2
	Sempre	4	5	5	5	4	3	5	3
	Poucas vezes	2	3	3	4	3	2	4	3
	Sempre	3	5	4	4	4	4	5	4
	Poucas vezes	2	2	2	3	2	2	4	2
	Sempre	3	4	4	4	3	4	4	3
	Sempre	3	5	4	5	4	2	5	1
	Poucas vezes	1	4	3	5	2	2	4	3
	Sempre	2	5	4	5	4	3	5	3
	Poucas vezes	2	4	4	5	3	3	5	4
	Sempre	2	4	2	4	3	4	3	4
	Sempre	2	5	4	5	5	4	5	3
	Sempre	4	4	4	4	4	2	4	4
	Sempre	2	4	2	5	2	2	4	3
	Sempre	3	5	4	4	3	2	4	3
	Sempre	2	4	2	4	2	2	4	3
	Sempre	2	4	2	5	4	2	4	5
	Sempre	5	5	5	4	5	4	5	4
	Poucas vezes	3	4	3	2	4	4	4	4
	Poucas vezes	3	4	2	4	2	3	4	1
	Sempre	3	5	4	4	4	4	4	4
	Sempre	3	4	2	4	2	4	4	2
	Poucas vezes	4	5	5	4	4	4	5	3
	Poucas vezes	4	4	4	3	4	4	4	2
	Poucas vezes	1	3	1	3	2	1	3	2
	Poucas vezes	4	4	3	2	2	3	4	2
	Nunca	3	3	2	4	4	2	5	2
	Sempre	4	3	2	3	2	4	4	3
	Sempre	2	4	4	4	3	4	4	2
	Sempre	2	5	5	4	3	1	4	3
	Sempre	3	4	4	4	4	5	5	4
	Poucas vezes	2	4	2	2	2	2	4	4
	Sempre	4	4	4	4	4	4	4	3
	Poucas vezes	2	4	2	4	3	2	4	3
	Poucas vezes	2	4	2	4	3	4	4	1
	Sempre	4	5	1	4	3	5	5	4
	Poucas vezes	2	5	4	5	5	4	5	2
	Sempre	1	5	4	5	5	4	4	2
	Poucas vezes	4	4	4	4	4	4	4	2
	Sempre	5	5	5	5	5	5	5	5
	Poucas vezes	3	4	2	2	2	3	4	2
	Sempre	4	4	4	4	4	3	4	2
	Sempre	3	4	3	4	4	3	5	3
	Poucas vezes	2	4	4	5	2	4	5	3
	Poucas vezes	2	4	2	4	2	3	4	3
	Poucas vezes	3	5	3	1	2	1	3	2
	Poucas vezes	4	4	3	4	3	4	3	3
	Poucas vezes	2	4	3	5	2	4	5	2

	Poucas veze:	4	5	4	4	3	4	5	3
	Sempre	3	5	2	4	3	2	4	1
	Poucas veze:	1	4	2	2	1	1	2	1
	Sempre	3	5	5	4	3	3	4	3
	Sempre	4	5	5	4	5	5	5	4
	Poucas veze:	2	4	4	3	2	5	5	1
UPA	ᵓoucas veze:	2	1	4	2	1	2	4	2
	Poucas veze:	4	4	3	2	3	4	4	3
	Poucas veze:	3	3	3	3	2	2	4	3
	Poucas veze:	4	3	3	3	2	2	4	2
	Sempre	1	4	2	4	4	1	4	3
	Sempre	4	5	4	5	4	5	5	4
	Sempre	3	4	4	4	3	4	5	3
	Sempre	3	4	4	5	4	4	4	4
IVERSITÁRIC	Sempre	4	5	5	5	5	4	5	3
	Sempre	4	5	4	3	2	4	5	3
	Poucas veze:	1	4	1	4	2	1	4	3
	Sempre	2	4	3	4	1	4	3	2
	Sempre	4	5	4	4	4	2	4	3
	Sempre	4	5	4	5	4	4	5	4
	Sempre	5	5	5	5	4	4	5	5
	Poucas veze:	4	4	2	4	3	4	4	2
	Poucas veze:	4	4	4	4	3	4	4	3
	Sempre	3	3	2	2	3	1	4	3
	Poucas veze:	3	4	4	4	3	4	5	4
	Poucas veze:	2	4	3	4	3	2	2	2
	Sempre	3	4	4	3	2	2	4	2
	Sempre	3	4	3	4	4	4	5	3
	Poucas veze:	2	4	2	4	2	4	4	4
	Sempre	2	5	3	5	4	3	5	4
	Sempre	2	4	5	4	2	3	5	3
ital Felicio R	Sempre	5	5	5	5	5	5	5	3
	Poucas veze:	4	4	2	2	4	3	4	5
	Poucas veze:	3	4	3	4	3	1	4	1
	Sempre	4	4	4	4	4	4	4	3
	Poucas veze:	2	2	2	4	2	2	4	2
	Poucas veze:	2	4	3	2	2	4	5	3
	Poucas veze:	2	4	3	4	2	3	4	3
	Sempre	4	4	3	3	3	4	4	2
	Sempre	2	4	3	4	3	4	4	2
Hospital	Sempre	5	5	5	5	4	1	5	5
	Sempre	3	5	3	5	2	2	5	1
	Sempre	5	5	4	4	3	3	5	3
	Poucas veze:	4	5	3	5	3	4	5	3
	Nunca	4	4	3	3	4	4	4	2
	Sempre	3	5	5	5	5	4	5	3
	Nunca	1	1	1	3	1	1	4	3
	Sempre	3	4	4	3	4	3	4	4
	Poucas veze:	2	4	2	5	2	4	5	4
	Poucas veze:	2	4	2	4	3	3	4	3
	Poucas veze:	3	4	4	5	3	3	5	4
	Sempre	5	5	4	5	5	5	5	5
	Sempre	2	5	5	3	4	3	5	5
	Sempre	5	5	5	2	3	4	5	5
	Sempre	4	4	3	4	3	4	4	4
	Poucas veze:	2	5	4	5	3	2	4	5
	Sempre	2	5	4	4	5	4	5	4
	Sempre	4	5	5	5	4	5	4	3
	Sempre	2	5	5	3	3	4	4	1

ı de ginástic	Poucas veze:	2	3	2	3	2	2	4	3
	Poucas veze:	4	4	4	4	4	3	4	2
	Poucas veze:	1	2	2	2	2	2	2	3
	Sempre	5	5	3	3	3	3	5	3
	Poucas veze:	3	4	3	3	3	3	4	3
	Sempre	4	4	2	4	3	2	4	2
ital geral de	Poucas veze:	2	4	3	4	4	2	5	1
	Sempre	2	4	2	4	2	2	3	2
	Poucas veze:	3	4	5	4	4	4	5	4
≥atendiment	Nunca	1	4	1	2	1	5	5	1
	Sempre	4	4	4	5	3	3	5	4
	Poucas veze:	4	5	4	4	3	3	5	4
	Poucas veze:	1	3	2	4	1	1	5	3
	Sempre	2	5	3	4	2	3	4	3
	Poucas veze:	2	4	2	2	2	2	5	4
	Sempre	4	4	4	4	4	4	4	2
	Sempre	4	5	4	5	5	4	4	4
	Sempre	2	5	3	4	3	2	3	3
CONTA COM	Sempre	2	4	4	4	2	3	4	2
	Poucas veze:	1	5	1	4	1	4	5	1
	Sempre	4	4	4	2	4	4	4	2
	Poucas veze:	4	4	3	4	3	4	4	3
ıssiano Antô	Sempre	5	4	4	5	3	5	4	5
	Poucas veze:	3	4	2	3	3	3	3	1
	Poucas veze:	5	4	4	4	4	3	5	5
	Sempre	2	4	4	4	2	5	5	4
	Poucas veze:	1	3	1	2	1	1	3	2
	Sempre	4	4	4	3	4	4	4	3
	Poucas veze:	2	2	2	1	1	4	3	2
nagem em ι	Sempre	5	5	4	5	4	3	5	4
	Sempre	3	5	3	5	4	2	4	3
	Sempre	4	5	2	3	3	2	4	3
	Sempre	4	5	4	5	3	3	4	2
	Poucas veze:	2	4	3	4	2	3	4	3
	Sempre	3	4	2	2	2	2	4	4
	Sempre	2	4	3	4	2	1	3	3
	Sempre	3	4	4	3	3	3	4	2
	Poucas veze:	3	5	3	5	2	1	4	4
	Poucas veze:	3	4	4	4	3	2	4	3
	Sempre	5	5	4	4	5	5	5	5
	Sempre	4	5	5	5	4	4	5	3
	Sempre	2	4	3	4	3	2	4	3

AP1	AP2	AP3	AP4	CE1	CE2	CE3	CFH1	CFH2	CFH3
5	5	5	5	4	4	5	2	5	2
5	5	5	5	4	4	4	5	5	5
4	4	5	5	5	5	5	4	5	5
2	4	4	4	4	5	5	1	2	3
5	5	5	5	1	1	4	5	5	5
4	4	4	4	4	4	2	4	4	4
4	4	2	2	3	4	4	1	1	1
4	5	4	5	2	2	4	1	5	4
5	4	5	5	4	4	5	4	4	4
4	5	4	4	3	4	4	5	4	5
4	4	2	2	2	2	4	2	4	4
5	5	5	5	2	3	4	5	5	5
4	3	4	4	1	4	4	2	4	4
5	4	4	4	4	4	4	2	4	4
4	4	3	3	2	4	4	2	4	4
4	4	4	4	4	4	5	4	4	4
4	4	3	4	5	5	5	2	3	4
4	5	4	5	2	5	5	4	5	4
1	2	1	2	1	5	4	2	3	3
4	4	4	4	4	2	4	2	4	4
5	5	5	5	4	4	5	2	4	4
4	4	4	4	3	4	4	3	4	4
4	4	4	4	5	3	4	4	4	4
5	4	5	5	2	4	4	4	4	4
5	4	5	5	5	5	5	3	5	5
3	5	4	4	2	3	2	4	5	4
5	5	4	4	3	4	5	5	5	5
4	4	5	4	5	5	5	5	5	4
4	4	4	2	5	5	5	5	4	4
4	4	4	4	2	3	3	4	4	4
4	4	4	4	5	5	5	3	4	4
5	5	5	5	3	4	4	4	4	4
5	5	4	4	3	3	4	4	4	5
5	5	5	5	4	4	4	5	5	5
5	5	5	5	4	4	4	2	5	4
5	4	4	4	4	4	4	2	5	4
4	4	3	3	3	3	4	4	5	5
5	4	5	5	5	5	5	1	5	5
4	4	4	5	2	2	4	4	4	4
4	4	5	4	5	5	5	1	4	3
4	4	4	4	2	3	5	4	4	3
5	5	5	5	4	5	5	4	5	5
3	4	3	3	2	4	4	4	4	4
5	5	5	5	3	4	5	4	4	4
4	4	4	4	2	4	4	2	5	4
4	4	4	4	4	4	4	4	4	4
5	4	4	4	4	3	3	5	5	4
4	4	4	4	4	4	5	4	4	4
5	3	5	5	2	2	4	4	5	4
5	5	5	5	5	5	5	2	4	4
4	4	4	4	4	4	4	2	4	2
3	4	3	2	4	2	4	4	3	2
4	4	4	4	4	4	5	2	4	3
5	5	5	5	3	3	4	4	4	5
5	5	5	5	4	4	4	5	5	4
4	4	4	4	4	5	5	4	3	4
4	5	5	5	4	4	4	4	5	4
5	5	5	5	1	4	5	4	4	4

5	4	5	4	5	5	5	4	5	5
3	5	3	4	5	5	5	2	5	4
5	5	5	5	4	4	5	4	5	5
4	4	3	3	4	4	4	3	4	2
5	4	5	5	2	1	5	4	5	3
5	4	5	5	3	3	4	2	5	4
4	4	4	4	2	2	3	2	3	4
5	5	4	4	3	3	3	4	3	4
5	5	3	5	1	4	4	5	5	5
5	5	4	4	2	4	5	1	4	1
5	5	5	5	4	4	5	2	4	4
4	4	4	4	5	5	5	4	4	4
5	5	5	4	4	4	5	3	4	4
4	3	4	4	3	3	3	3	4	3
5	5	5	5	4	4	5	4	5	5
2	4	2	2	2	4	4	2	4	3
4	4	4	4	4	4	4	4	4	4
4	5	1	5	3	4	5	4	3	5
4	4	4	4	4	2	5	2	5	3
5	5	5	5	5	5	5	4	2	1
2	4	4	4	5	5	5	3	4	4
5	4	5	5	4	4	4	3	4	3
5	5	5	5	2	2	4	4	4	5
4	4	4	4	2	4	4	2	4	4
4	4	4	3	2	2	4	2	4	4
5	4	5	4	5	5	5	2	4	4
5	4	2	4	4	5	5	4	5	5
5	4	4	5	5	5	5	2	4	2
5	5	5	5	2	2	5	5	5	5
4	4	4	4	4	4	4	4	5	5
4	4	2	3	2	5	5	4	4	4
5	4	4	4	4	4	4	5	5	5
4	4	4	4	4	4	5	2	5	4
4	4	4	4	2	2	4	3	1	3
3	4	4	4	4	5	5	4	4	4
2	2	3	2	4	5	5	4	2	3
3	4	4	4	4	4	4	2	3	3
4	4	4	4	2	2	5	2	2	4
4	5	4	3	4	4	4	5	5	5
4	4	5	5	4	4	4	4	5	5
4	5	5	5	2	3	4	1	4	4
3	5	3	4	2	3	4	4	5	5
4	3	3	3	4	4	4	5	4	4
4	3	4	4	2	4	4	4	4	4
2	4	2	2	2	2	3	4	3	4
4	5	4	3	3	3	3	5	3	4
5	5	4	4	1	4	4	5	5	5
5	5	5	5	5	5	5	5	5	5
5	5	5	5	4	4	5	3	4	3
3	4	3	4	3	2	4	1	5	4
5	5	5	5	4	4	5	2	5	5
5	5	5	4	4	4	5	3	3	4
4	4	4	4	4	4	4	1	5	4
4	4	4	4	3	3	3	3	4	3
5	5	5	5	1	3	5	2	5	4
4	4	3	4	4	4	5	1	3	3
4	4	2	3	4	4	2	1	4	2
4	4	4	4	4	4	4	4	4	4
4	4	4	4	2	4	4	4	5	4

5	5	5	4	4	4	4	5	5	5
4	4	4	5	5	5	5	2	4	4
5	4	1	4	5	5	5	1	2	2
2	4	2	3	2	5	5	3	4	4
4	5	4	5	1	3	5	5	5	4
4	5	5	4	5	5	5	4	4	5
3	2	3	3	2	2	5	2	4	2
4	5	4	4	4	4	4	1	4	4
3	3	3	3	4	3	3	4	5	4
2	4	3	3	5	5	5	4	3	4
4	4	4	4	2	4	2	2	4	4
5	5	5	5	2	4	5	4	5	5
4	3	5	5	4	4	5	2	5	4
5	5	5	4	4	4	4	5	5	5
5	5	4	4	1	1	4	2	4	4
5	4	4	4	2	3	4	5	5	4
4	4	4	4	4	4	4	4	4	4
4	4	5	4	3	1	4	2	5	4
4	4	4	4	4	4	5	4	4	4
5	4	5	4	4	1	5	4	4	2
5	5	5	5	2	3	4	5	5	5
4	5	4	3	4	5	4	5	4	4
4	4	3	3	4	4	4	4	3	4
4	5	3	4	2	2	3	2	4	3
5	4	5	4	1	2	3	5	4	4
3	5	3	4	2	2	3	5	4	4
4	4	3	4	4	4	4	4	4	4
5	4	5	4	3	3	4	2	5	5
4	4	4	4	2	4	5	4	4	4
5	5	4	5	2	2	4	4	4	5
5	5	5	5	3	1	4	4	4	4
5	5	5	5	4	4	5	5	5	5
4	5	4	2	4	4	4	5	5	4
3	4	4	3	3	3	4	2	4	4
4	4	4	4	4	4	4	4	4	4
2	4	4	4	4	5	5	4	2	4
4	4	4	4	4	4	5	5	4	4
4	3	4	4	4	4	4	2	4	4
4	5	4	4	2	3	4	2	4	4
4	5	4	5	4	4	5	4	4	3
5	3	1	5	5	5	5	5	5	5
5	4	5	5	3	5	5	2	5	5
5	5	5	5	4	3	5	3	5	4
5	4	5	5	3	5	5	2	5	5
4	4	4	4	4	4	4	2	5	4
5	4	5	5	4	4	4	2	4	4
1	3	2	1	3	3	3	3	3	3
3	4	4	4	1	2	2	4	4	4
5	5	3	3	4	4	4	5	5	4
4	4	3	4	3	3	3	4	4	4
4	4	4	4	4	5	4	3	3	4
5	5	5	5	4	4	4	5	5	5
5	5	5	5	5	5	5	4	5	5
5	5	5	5	3	4	4	5	5	5
4	4	4	4	3	3	4	4	4	4
5	5	4	5	2	2	3	3	5	4
5	5	5	4	5	5	5	4	5	5
5	5	5	5	3	4	5	4	5	5
5	5	5	5	5	5	5	2	5	5

4	3	3	4	4	4	4	3	4	4
4	4	2	4	4	4	4	4	5	4
2	2	2	2	4	4	4	2	3	2
5	5	5	5	3	3	4	3	5	4
3	3	1	2	4	4	4	2	2	2
4	4	4	4	4	4	4	4	5	5
5	4	4	3	5	5	5	1	4	4
3	4	4	4	4	4	4	3	4	4
4	5	4	5	4	4	4	3	4	4
5	5	5	3	1	5	5	4	5	5
5	4	4	3	3	4	4	2	3	3
5	5	4	4	4	4	4	3	4	4
2	4	2	4	4	5	4	2	4	4
5	5	4	4	4	4	4	3	4	4
2	2	4	2	2	2	2	2	4	4
4	4	4	4	2	4	4	3	4	3
4	5	4	4	4	4	5	4	5	4
5	5	5	5	3	3	4	2	4	3
5	5	4	4	2	2	4	1	1	2
1	5	4	4	4	5	5	1	4	4
4	4	4	4	4	4	5	2	3	5
4	4	4	5	4	4	5	4	4	4
5	4	5	4	4	4	4	2	4	4
3	2	2	3	4	5	5	4	4	4
3	5	5	4	4	5	5	4	5	4
5	5	4	5	4	4	4	5	4	5
3	2	2	2	1	2	2	4	3	2
4	4	4	4	2	4	4	4	4	4
2	3	1	2	2	3	4	2	4	2
5	4	4	5	3	2	4	5	4	4
4	5	5	5	4	4	4	5	5	5
4	5	4	4	4	3	5	1	5	3
5	4	5	5	4	4	5	4	5	4
5	4	5	5	5	5	5	3	5	4
4	4	4	4	4	3	3	4	4	4
5	4	4	5	3	4	4	4	5	5
4	4	4	4	4	4	4	3	4	4
5	3	5	5	5	5	5	2	4	3
4	5	5	3	4	4	4	3	4	4
5	5	4	5	2	4	4	5	5	5
5	5	5	4	4	5	5	3	5	5
5	5	5	5	4	5	5	4	4	4

CFH4	CS1 - Não invertido	CS2 - Não invertido
5	4	4
5	3	2
5	2	3
4	4	4
5	1	1
5	3	3
5	4	4
5	2	4
5	1	3
5	1	1
4	2	4
5	1	3
5	2	4
5	5	3
5	4	2
5	5	2
5	4	4
4	3	2
4	3	4
4	2	3
5	2	4
5	2	4
5	2	3
5	2	3
5	2	4
5	4	2
5	2	3
4	2	2
5	2	1
5	2	1
5	2	3
5	2	2
5	2	1
5	4	1
5	2	2
5	1	3
3	1	1
5	1	3
4	2	2
4	1	2
5	2	4
5	1	2
3	2	3
5	1	4
5	1	2
4	2	2
5	4	1
5	1	1
5	2	2
5	1	4
4	2	3
5	2	3
4	2	4
5	2	4
5	2	2
2	4	3
5	1	2
5	1	4

5	2	3
5	2	4
5	1	3
5	2	5
5	3	3
5	3	3
4	4	3
4	1	3
5	1	2
4	3	4
5	2	4
5	1	2
5	2	4
4	3	3
5	4	2
4	2	3
5	2	3
5	1	5
5	1	4
5	2	4
5	1	2
5	1	3
5	2	2
5	2	2
5	4	2
4	3	4
5	3	3
5	2	3
5	1	4
5	2	2
5	2	2
5	3	3
5	2	3
2	3	2
5	2	5
4	2	2
3	3	3
4	4	5
5	1	3
5	2	2
4	2	3
5	2	2
5	4	2
4	2	3
4	2	3
4	1	3
5	2	3
5	1	2
5	1	2
5	3	3
5	4	4
5	3	4
5	4	4
5	2	2
5	2	2
4	1	4
4	2	4
4	3	5
4	2	2

4	2	3
5	3	4
5	2	4
5	4	3
5	4	5
5	4	5
5	3	5
3	1	2
5	2	2
4	2	2
4	2	2
5	2	3
5	2	3
5	2	3
5	1	4
5	2	5
5	1	3
5	4	2
4	4	4
5	1	2
5	3	3
4	3	2
4	2	4
5	2	2
5	1	3
5	2	3
5	2	3
5	2	4
2	2	2
5	1	2
5	1	3
5	5	3
5	3	2
4	1	3
4	3	4
5	4	4
5	2	3
5	4	4
5	2	4
5	1	4
5	1	2
5	2	4
5	3	4
5	1	3
2	1	3
5	4	4
2	3	3
3	4	3
5	2	3
4	3	4
3	1	2
5	1	2
5	1	4
5	2	3
4	1	3
3	2	4
5	2	3
5	2	4
5	1	4

3	3	2
5	2	3
4	2	3
5	2	4
4	2	3
5	2	2
5	1	2
5	2	3
5	2	2
5	1	5
5	2	3
4	1	3
4	4	4
5	2	3
5	3	2
4	4	3
5	4	2
5	3	3
5	2	3
5	4	4
4	2	4
5	3	3
2	2	4
3	2	3
4	2	3
5	2	3
4	4	3
4	2	2
5	2	3
5	2	1
5	1	2
5	2	1
5	2	4
5	1	3
5	1	1
5	3	2
5	5	4
5	2	2
5	4	4
5	2	2
5	1	3
5	2	2
